# Adaptation to elevated CO_2_ in different biodiversity contexts

**DOI:** 10.1038/ncomms12358

**Published:** 2016-08-11

**Authors:** Elizabeth J. Kleynhans, Sarah P. Otto, Peter B. Reich, Mark Vellend

**Affiliations:** 1 Department of Zoology and Biodiversity Research Centre, University of British Columbia, 2212 Main Mall, Vancouver, British Columbia, Canada V6T 1Z4; 2 Department of Forest Resources, University of Minnesota, 1530 Cleveland Avenue North, St Paul, Minnesota 55108, USA; 3 Hawksbury Institute for the Environment, Western Sydney University, Penrith, New South Wales 2751, Australia; 4 Département de biologie, Université de Sherbrooke, 2500 Boulevard de l'Université, Sherbrooke, Québec, Canada J1K 2R1

## Abstract

In the absence of migration, species persistence depends on adaption to a changing environment, but whether and how adaptation to global change is altered by community diversity is not understood. Community diversity may prevent, enhance or alter how species adapt to changing conditions by influencing population sizes, genetic diversity and/or the fitness landscape experienced by focal species. We tested the impact of community diversity on adaptation by performing a reciprocal transplant experiment on grasses that evolved for 14 years under ambient and elevated CO_2_, in communities of low or high species richness. Using biomass as a fitness proxy, we find evidence for local adaptation to elevated CO_2_, but only for plants assayed in a community of similar diversity to the one experienced during the period of selection. Our results indicate that the biological community shapes the very nature of the fitness landscape within which species evolve in response to elevated CO_2_.

## Abstract (web summary)

How do species adapt to environmental change when living with different kinds of competitors? Through a reciprocal transplant experiment, the authors show that competitive community alters the nature of selection so that species adapt to elevated CO_2_ in different ways in varying community contexts.

## Graphical abstract

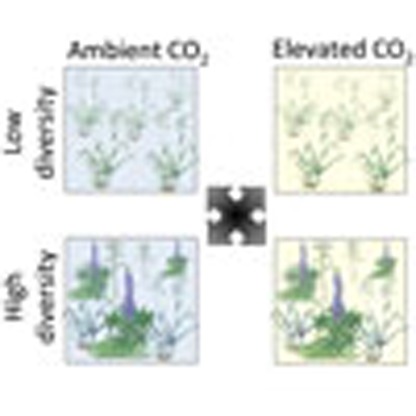

Species do not evolve in isolation but within a community of interacting species. While some evidence exists for the impact of predator–prey^1^,^2^,^3^ or host–parasite interactions^4^ on adaptive evolution, we lack experimental data on the impact of competition on adaptation in natural systems. Laboratory and mesocosm studies have found contrasting results for how competition influences adaptation. Competition can inhibit adaptation, as found in algal cultures (*Chlamydomonas reinhardtii*) evolving to elevated CO_2_ (ref. 5). Similarly, adaptive diversification to habitat heterogeneity in *Pseudomonas flourescens* was prevented in the presence of interspecific competitors, as these competitors exclude intraspecific variants^6^. Competition can also alter the nature of selection^2^,^7^,^8^. For instance, increased water temperature caused the zooplankton *Daphnia magna* and *D. pulex* to evolve faster growth in the absence of competition but to evolve a larger size at maturity in the presence of competitors and predators^2^. Similarly, bacterial species adapted differently to a novel environment when grown alone or with other bacteria species^7^. In plants, the presence, composition, and diversity of competing species shows tremendous spatial variation^9^, can have a major impact on individual performance^10^,^11^, and thus might have an important influence on species' adaptation to environmental change. Yet, how biotic community context alters how species adapt to environmental change in natural field settings remains entirely unknown.

To address this knowledge gap, herein we report on an investigation of the impact of prairie grassland communities on the evolutionary response to elevated CO_2_ over 14 years in the Biodiversity Carbon dioxide and Nitrogen experiment (BioCON) at the Cedar Creek Ecosystem Sciences Reserve (Minnesota, USA)^12^. To determine how the surrounding biological community influences a species' ability to evolve in response to abiotic change, we focused on very different community structures: monoculture versus high diversity. By focusing on the presence or absence of interspecific competitors, we increased our power to detect the impacts of the surrounding species diversity on evolution. We tested four possible scenarios by which species diversity might affect the evolutionary responses of a focal species to abiotic environmental change.

The first scenario is that species diversity has no effect on adaptation to abiotic environmental change (Fig. 1a,b). If selective pressures exerted by changing abiotic conditions overwhelm those from the biotic community, species diversity should have no impact on local adaptation to abiotic change. Statistically, the response to selection (fitness of plants evolved under elevated CO_2_ (eCO_2_) minus fitness of plants evolved under ambient CO_2_ (aCO_2_)) should be predicted only by the change in CO_2_ environment (ΔCO_2_), regardless of species diversity (Fig. 1a,b).

The second scenario is that species diversity constrains adaptation to abiotic environmental change (Fig. 1c,d). When grown with more species, local adaptation might be reduced because competition for space and resources results in smaller effective population sizes of each competing species, reducing standing genetic variation^13^,^14^ and the rate at which new mutations arise^15^. In addition, with more species in a community one species may, by chance, possess traits that pre-adapt it to the new niche(s) created by the changing environment^15^. This pre-adapted species will increase in abundance, creating more competition and resulting in a further decline in abundance, and therefore ability to adapt, in the other species^14^,^16^,^17^. Under this scenario, local adaptation to elevated CO_2_ should be more evident for plants that experienced selection in a species-poor community (Fig. 1c) than in a species-rich community (Fig. 1d), regardless of the diversity of the community into which the plants were transplanted (the ‘assay' community). Statistically, the response to selection should be predicted by a three-way interaction between the CO_2_ selection environment (CO_2_^sel^), the change in CO_2_ environment (ΔCO_2_), and diversity of the selection environment (div^sel^).

The third scenario is that species diversity promotes adaptation to abiotic environmental change (Fig. 1e,f). A more homogeneous environment caused by low species richness might select for and maintain fewer genotypes, reducing genetic diversity. Likewise, high species richness might increase environmental heterogeneity, thereby maintaining greater genetic variation and adaptive potential^10^,^18^. This scenario predicts that a focal species should adapt faster to abiotic change (for example, eCO_2_) when selection is experienced in a species-rich community. As with the second scenario, a significant CO_2_^sel^ × ΔCO_2_ × div^sel^ interaction should support this scenario, except with the opposite relationship to diversity (Fig. 1e,f).

The forth scenario is that species diversity changes the fitness landscape (Fig. 1g,h). The biological community may act to modify the selection environment created by eCO_2_ thereby changing the shape of the fitness landscape. That is, the surrounding community may act like a prism, transforming an applied selective pressure into the selective pressure actually experienced by a focal species^2^,^7^,^19^. For example, increased CO_2_ might select for faster growth, resulting in selection for more efficient nitrogen use in a species-poor community but not in a species-rich community that includes nitrogen-fixing plants^20^. As another example, belowground microbial biomass has been found to decline with eCO_2_ in species-poor communities but to rise in species-rich communities^21^, thereby potentially altering the supply and abundance of various nutrients to plants^22^ in different biotic and abiotic contexts to which plants must locally adapt. Whatever the mechanism, this scenario predicts local adaptation to eCO_2_ when fitness is assessed in a community with similar diversity as the community in which selection occurred (Fig. 1g,h). Statistically, this scenario should be supported by a significant interaction between CO_2_ selection environment (CO_2_^sel^), change in CO_2_ environment (ΔCO_2_), and change in diversity (Δdiv).

We tested these predictions using a reciprocal transplant experiment (Fig. 2) involving *Poa pratensis* (Kentucky bluegrass), a species widespread and abundant across several continents, and one of the more common species in BioCON^23^. We collected seeds from plots that had been exposed for 14 years to ambient or elevated (ambient+180 p.p.m.) concentrations of CO_2_ in species-poor (monoculture) or species-rich (16 species) grassland communities^12^. We transplanted individuals with all four ‘histories' into all four of these treatment combinations. Consistent with the fourth scenario, we find that the biological community alters the fitness landscape in elevated CO_2_, so that local adaptation is observed primarily when species are grown in a community similar to the one in which they were previously selected.

## Results

### Results for biomass production

**Figure 1:**
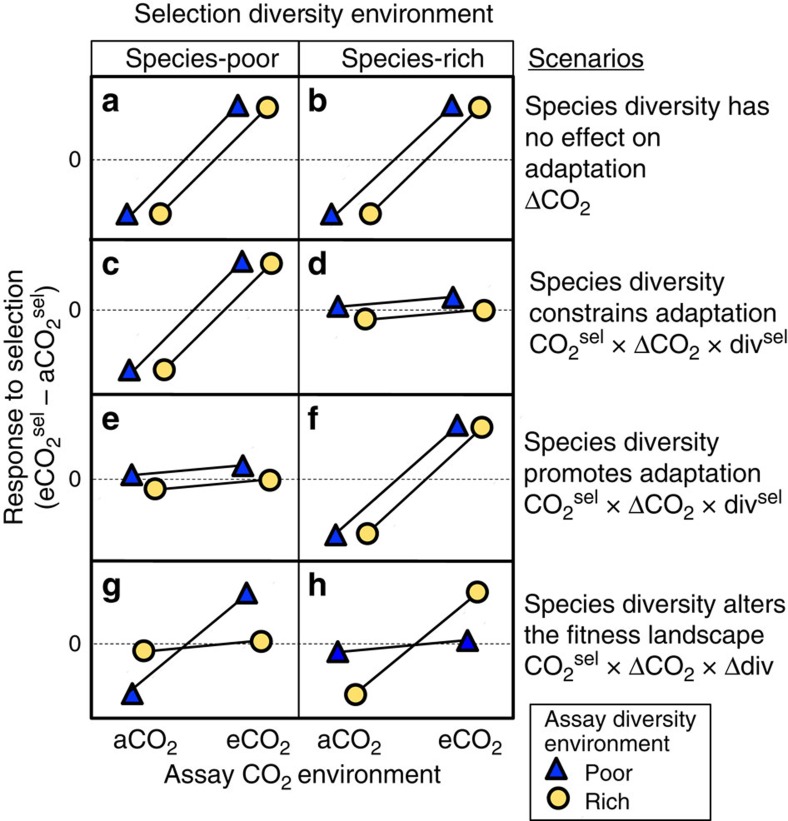
The hypothetical influence of species richness on adaptation of plants to elevated CO_2_. The response of a focal species to selection in a given context can be quantified as the difference in performance (for example, biomass or fitness) between plants originating from elevated CO_2_ (eCO_2_) plots and those from ambient CO_2_ (aCO_2_) plots (*y* axis). (**a**,**b**) If species diversity has no effect on adaptation, local adaptation to eCO_2_ should be similar in species-poor and species-rich communities regardless of the species richness of the assay environment. (**c**,**d**) If species diversity constrains adaptation, an evolutionary response to eCO_2_ should be more evident for plants that experienced selection in a species-poor community than in a species-rich community, regardless of the species richness of the assay environment. (**e**,**f**) If species diversity promotes adaptation to eCO_2_, then plants that experienced selection within a species-rich community may show greater fitness in eCO_2_ regardless of assay species richness. (**g**,**h**) If species diversity alters the fitness landscape in response to CO_2_, then plants may only show improved fitness in eCO_2_ when planted back into a community of similar richness to the one in which they experienced selection. Each scenario can be represented by a particular statistical term, shown on the right.

We found that *P. pratensis* locally adapted to the CO_2_ environment, but only when the diversity of the community was the same in the past ‘selection' and current ‘assay' environment. That is, the term ΔCO_2_ was statistically significant for aboveground (F_1, 306.6_=7.8, *P*=0.006) and total biomass (F_1, 302.9_=4.1, *P*=0.04) (Supplementary Table 1) when plants were selected and assayed in the same diversity treatment (species-poor to species-poor or species-rich to species-rich) (Supplementary Fig. 1). However, if adaptation to CO_2_ was assessed by averaging the results from both species-rich and species-poor assay plots, local adaptation was not statistically detectable for any measure (Supplementary Tables 2 and 3; Supplementary Fig. 2). We thus find that the local adaptation does not depend solely on the community context during past selection but also depends on the current assay environment, leading us to reject scenarios one to three (Fig. 1).

Local adaptation was only seen when we took into account both the diversity of the selection environment and the assay environment. This indicates support for the fourth scenario in which species diversity alters the fitness landscape (Fig. 1g,h): plants exhibited adaptation to eCO_2_, but only when assayed in the community context in which selection occurred (Fig. 3a–c). For example, the response of aboveground biomass to selection in eCO_2_ (Fig. 3a) was positive in species-poor environments (left) but chiefly when assayed in species-poor environments (dark triangles). Similarly, plants from species-rich environments showed adaptation to eCO_2_ but only when assayed in species-rich environments (circles on the right). As a result, when performing a full statistical analysis including community diversity in both selection and assay environments, there were significant three-way CO_2_^sel^ × ΔCO_2_ × Δdiv interactions for aboveground (F_1,689.1_=5.8, *P*=0.016), belowground (F_1,690.3_=4.2, *P*=0.041) and total biomass (F_1,684.3_=4.2, *P*=0.039) (Supplementary Table 3), with increased adaptation to eCO_2_ when the community context remained the same between selection and assay environments (Fig. 3a–c). These significant interactions were consistent with plants demonstrating a ‘home' plot advantage when assayed in a biotic and abiotic environment similar to the one in which they experienced selection (Supplementary Fig. 3a–c).

### Results for survival and inflorescence production

The high survival of individuals across treatments (>80% survival) and the lack of flowering within species-rich plots (only 9% of individuals produced inflorescences) reduced our power to analyse these fitness components (Supplementary Fig. 3d). Although not significant, the direction of the results from an Aster analysis^24^ of survival and inflorescence production were consistent with the fitness landscape scenario (Fig. 3d; Supplementary Table 5).

### Analysis with selection and assay environments

As an alternative statistical approach, we also analysed the data by treating the selection and assay environments (CO_2_^ass^ and div^ass^) (not the change in environments) as fixed factors (Supplementary Tables 4 and 6). This approach has reduced statistical power because the fourth scenario, the fitness landscape scenario, must be tested via a four-way interaction (CO_2_^sel^ × CO_2_^ass^ × div^sel^ × div^ass^), while the second and third scenarios are tested via three-way interactions (CO_2_^sel^ × CO_2_^ass^ × div^sel^). Nevertheless, these results were also consistent with the fitness landscape scenario (Supplementary Tables 4 and 6). Analysing the data in this alternative way aided in teasing apart immediate responses to the environment (that is, ‘plastic' responses) from evolutionary changes. Indeed, we also detected strong plastic responses, with greater biomass for plants assayed in species-poor plots and for plants assayed in eCO_2_ (Supplementary Tables 4 and 6; Supplementary Note 1).

## Discussion

Our study contrasted four scenarios for how the diversity of the surrounding neighbourhood community could impact evolutionary change in a focal species. Overall our results were most consistent with the scenario in which species diversity alters the fitness landscape (Fig. 1g,h) and indicate that while adaptation to eCO_2_ confers a performance advantage in both community contexts, the advantage does not transfer directly across community contexts. Studies examining the effect of eCO_2_ on plants and communities have found that eCO_2_ typically accelerates plant growth, alters plant tissue chemistry^25^, increases plant biomass and reduces evapotranspiration^26^. These changes result in increased soil water content^27^, altered belowground microbial diversity^28^ and modified nutrient cycling. The nature of these changes, however, is likely to depend on the surrounding community. Indeed, increasing plant diversity also reduces soil moisture^27^, increases microbial community diversity^21^ and increases nutrient cycling^29^. These changes are expected to interact^21^ to shape the selective environment in which a plant grows. Since competition exerts strong selective pressures, we suggest that it is the combined effect of altered abiotic environment and changed competitive interactions shaping the fitness landscape that drive our result. A similar perspective is that, as mutations arise, their pleiotropic effects generate different selective trade-offs depending on the surrounding community, altering which mutations can spread (which is another way of saying that the community shapes the fitness landscape experienced by new mutations).

Our finding that adaptation to eCO_2_ does not transfer across community contexts may have important implications for understanding how species will respond at both small and large scales to rising global CO_2_ levels caused by fossil-fuel emissions. At small scales the composition of communities can vary tremendously due to aspect, slope, soil, and so on, and the evolutionary response of a metapopulation to a common selective pressure (for example, changing CO_2_) might be experienced in different ways at different sites, with the local community altering the selection experienced. With high gene flow between populations, adaptation to rising CO_2_ levels may be hampered as a consequence of maladaptation to the biotic environment. At larger scales, species are likely to shift their range boundaries in response to climate warming and therefore to encounter novel community contexts^30^,^31^. If range shifts alter the biotic community, previous adaptations to the abiotic environment (for example, elevated CO_2_ or temperature) may no longer improve fitness when in a different community context.

To our knowledge, our study is the first experiment conducted in a natural field setting to test whether adaptation to an abiotic change in a macro-organism is impacted by community context. In plants, several studies have investigated the interaction between abiotic and biotic conditions on adaptation, but they either were not conducted in a natural field setting (that is, a pot experiment with a single competitor species^8^) or they focused on immediate phenotypic responses^32^,^33^,^34^,^35^. By performing a reciprocal transplant experiment of plants propagated under different biotic and abiotic conditions for 14 years, we can distinguish plastic from evolutionary responses in different community contexts.

Another contribution of this experiment is that it provides an additional compelling example of local adaptation at both a small spatial and temporal scale. Adaptation was observed in a perennial species in just 14 years to an important global change driver (eCO_2_) in a manner that depends on the community context at a microgeographic scale. This work thus contributes to the growing number of examples from field populations of local adaptation over short temporal^36^,^37^ or microgeographic^38^,^39^ scales.

There are some limitations to our study. First, the response to eCO_2_ was relatively modest, and responses to stronger agents of selection could be less or more contingent on biotic context. Second, there was limited scope for replication given the design of the original BioCON experiment; in particular, only two monoculture plots existed for *P. pratensis* under each CO_2_ treatment. Another consideration is that although *P. pratensis* reproduces predominantly asexually through rhizomatous growth or apomixis^40^, making selection within and among clones a likely mechanism of adaptation, on-going gene flow, through seed or pollen dispersal, from other BioCON plots or the surrounding prairie plant community (including *P. pratensis*) cannot be discounted (although this would reduce the likelihood of observing the selection responses that we did). We should also emphasize that we have compared only two types of communities, species-rich and species-poor. We do not know the extent to which our results are driven by diversity, *per se*, versus simply the presence of particular other species. However, an analysis of our *P. pratensis* aboveground biomass data versus percent cover of other species in the species-rich plots did not indicate that one species was driving our results (Supplementary Note 2; Supplementary Tables 7 and 8). Moreover, earlier studies in BioCON of effects of species-poor versus species-rich neighbourhoods on focal species show that resource competition and environmental stress amelioration (both via higher biomass related effects) are both enhanced by higher diversity^41^,^42^. Such effects may well have been at work in the current study. Finally, as with most studies of this nature, we cannot be certain that maternal effects were completely eliminated by clonal growth for 6 months in a common greenhouse environment. Thus, it remains possible that some of the evolutionary responses we have documented are in fact transgenerational maternal effects. Importantly, these limitations point largely to reasons one might expect not to find adaptation to CO_2_ in these plots over the past 14 years, but we did indeed find evidence for adaptation that fell clearly in line with one *a priori* prediction (and not the others).

Overall, our results indicate that the evolutionary response of a plant to elevated CO_2_ is manifested primarily when grown in the same type of biological community in which it evolved (species-rich or species-poor). This pattern supports the view that the selection imposed by a shift in the abiotic environment is experienced through the prism of surrounding species, altering the form of selection actually experienced by a focal species, consistent with the fitness landscape scenario. How often the biological community acts to change the selection experienced in altered environments can only be determined by additional empirical tests of these scenarios.

## Methods

### Sampling design

BioCON was initiated in 1997 and consists of six, 20 m-diameter circular rings, each with ∼66 2 × 2 m plant communities (that is, plots)^12^. In three randomly selected rings, atmospheric CO_2_ is elevated by ∼180 p.p.m. above ambient, using free air carbon dioxide enrichment (FACE) technology, the other three rings are maintained at ambient conditions. In each plot, 1, 4, 9 or 16 grassland species were initially seeded (12 g m^−2^ of seed partitioned equally among all species planted in a plot). The 16 species planted into BioCON are four C4 grasses (*Andropogon gerardii*, *Bouteloua gracilis*, *Schizachyrium scoparium*, *Sorghastrum nutans*), four C3 grasses (*Agropyron repens*, *Bromus inermis*, *Koeleria cristata*, *P. pratensis*), four nitrogen-fixing legumes (*Amorpha canescens*, *Lespedeza capitata*, *Lupinus perennis*, *Petalostemum villosum*), and four non-nitrogen-fixing forbs (*Achillea millefolium*, *Anemone cylindrica*, *Asclepias tuberosa*, *Solidago rigida*). The plots are maintained through regular weeding. Although BioCON also manipulated nitrogen (ambient and elevated), only plots exposed to ambient nitrogen were included in the current study.

*P. pratensis* (Poaceae), the focal species in our study, is a perennial, facultatively apomictic grass. It reproduces largely via asexually produced seeds^40^,^43^ or via tillers (ramets). Although native to Europe, *P. pratensis* is extensively naturalized in North America due to its use as a fodder and turf grass^44^.

**Figure 2:**
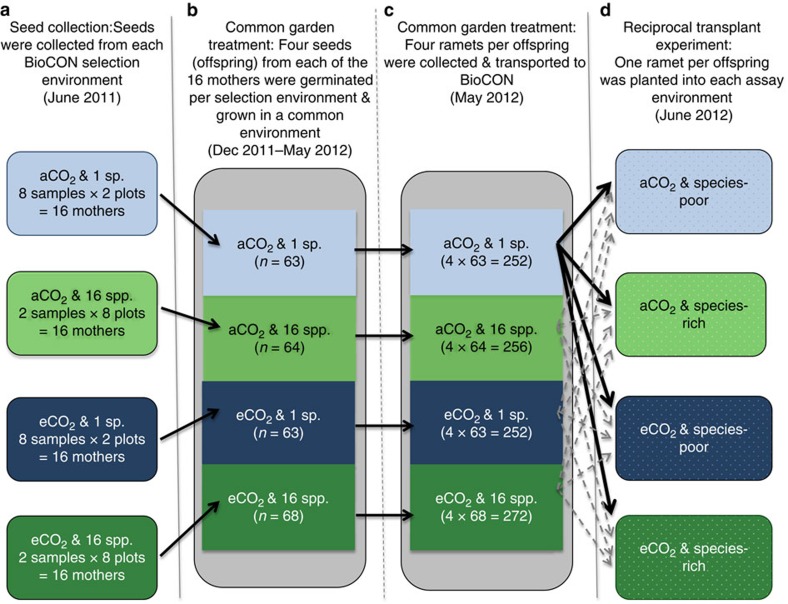
Steps involved in the transplant experiment. (**a**) *P. pratensis* seeds were collected from BioCON plots from different CO_2_ and diversity treatments. (**b**) Seeds were germinated in a common garden greenhouse environment to reduce maternal environmental effects. Values in brackets indicate the total number of germinated seeds per BioCON selection environment (departures from 64 due to failed germination or additional sampling are specified in the data table). (**c**) Four daughter ramets were sampled per germinated seed (offspring) in the greenhouse and transported back to BioCON for the transplant experiment. (**d**) One daughter ramet per offspring was placed into each of the assay diversity and CO_2_ environments (see text for additional details). 1 sp., *P. pratensis* monoculture BioCON plots; 16 spp., 16 species BioCON plots; species-poor, *P. pratensis* dominated species-poor plots; species-rich, species-rich plots (note that the latter two plots types describe the assay plots; Supplementary Methods 1).

**Figure 3:**
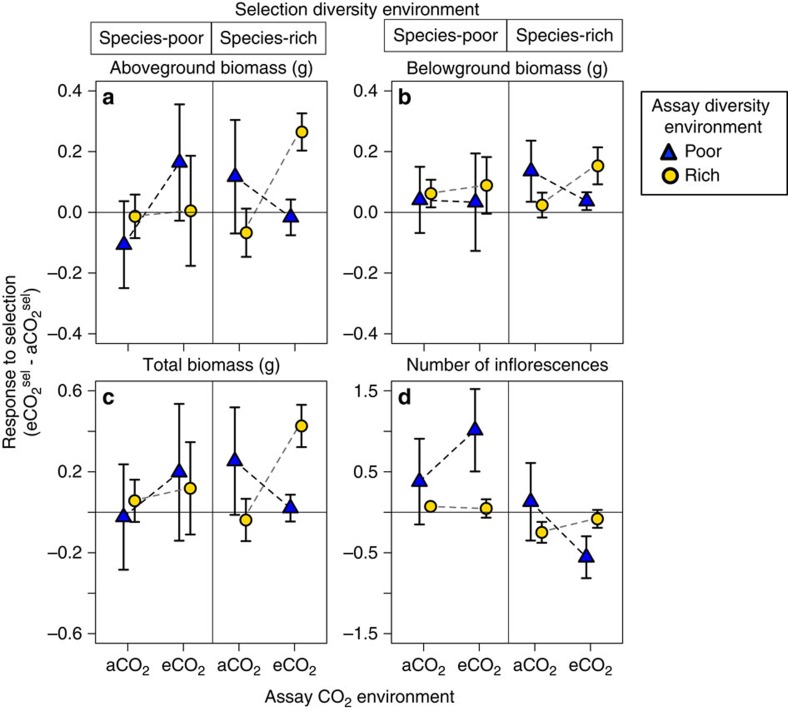
Response to selection under elevated versus ambient CO_2_ in species-poor and species-rich communities. For each assay CO_2_ environment we calculated the difference in (**a**) aboveground biomass, (**b**) belowground biomass, (**c**) total biomass in grams or (**d**) number of inflorescences (±1 s.e.m.) produced by plants that had previously experienced selection in eCO_2_ and in aCO_2_ from the raw data. Results are most consistent with the scenario that species diversity alters the fitness landscape (Fig. 1g,h).

To assess the impact of community diversity on adaptation to CO_2_, we conducted a reciprocal transplant experiment, with an initial six-month period of plant growth and vegetative reproduction in a common greenhouse environment to reduce maternal effects. In June 2011, we collected *P. pratensis* seeds from species-poor (monoculture) and species-rich (16 species) plots in aCO_2_ and eCO_2_ conditions (Fig. 2a). Four of the six BioCON rings contain a single *P. pratensis* monoculture plot (two rings with aCO_2_ and two with eCO_2_). Within these four rings, we sampled seeds from both the monoculture plot and 16-species plots. For each *P. pratensis* monoculture plot, we sampled eight evenly spaced individuals (that is, 2 rings × 1 plot × 8 mothers=16 mothers sampled per CO_2_ treatment). Each ring also contains four 16-species plots, from which we sampled two widely spaced individuals per plot (that is, 2 rings × 4 plots × 2 mothers=16 mothers sampled per CO_2_ treatment) (Fig. 2a).

### Growth in a common greenhouse environment

In order to reduce maternal environmental effects, all plants were subjected to a period of growth and vegetative reproduction in a common greenhouse environment. We broke seed dormancy by storing the seeds in a refrigerator (∼4 °C) in airtight containers with Drierite desiccant (to absorb moisture) for 5 months. In December 2011 we weighed all seeds and planted five per 656 ml pot (6.4 × 25.4 cm Deepots, model D40 H; Stuewe & Sons, Tangent, OR, USA), filled with potting soil at the University of British Columbia greenhouses. Twenty seeds collected from the same mother were planted. Seed weight (mean=9.6 mg per seed) was not statistically different between mothers or between CO_2_ or diversity treatments, suggesting that there were not substantial differences in maternal provisioning among treatments. After planting, the pots were randomized and placed on an unlit greenhouse bench and misted with water every 20 min. When the first seed within a given pot germinated, the pot was moved to 16 h of full spectrum light. Overall, 95% of pots had at least one germinated seed within 3 weeks of planting and within one week of one another. Only two pots completely failed (seeds never germinated and/or all seedlings died). The most robust seedling per pot was kept and the rest were removed, yielding a total of 258 plants (4 plants × 16 mothers × 2 CO_2_ treatments × 2 diversity treatments*—*2 that failed+4 that were mistakenly planted) (Fig. 2b). All plants were hand watered every second day with water enriched with fertilizer and all pots were randomized monthly.

The greenhouse plants never produced inflorescences, so we used vegetatively propagated daughter ramets (that is, ramets not from the central clump) for reciprocal transplantation back into BioCON. From 1–4 May 2012 we took five to six young ramets of approximately equal size from each *P. pratensis* plant raised in the greenhouse. These ramets were then planted into 164 ml (3.8 × 21 cm) cone-tainers (Ray-Leach, model SC 10 Super; Stuewe & Sons, Tangent, OR, USA) filled with potting soil and maintained under standard greenhouse conditions (Fig. 2c). On 27 May 2012 we removed all ramets from their cone-tainers and washed the roots free of soil. The roots were then wrapped in moist paper towel, and 1364 ramets were transported back to BioCON. The healthiest four ramets per plant were weighed and then randomly assigned to one of the four treatment groups (aCO_2_ species-poor; aCO_2_ species-rich; eCO_2_ species-poor; eCO_2_ species-rich) (Fig. 2d). No significant difference in biomass between ramets assigned to each treatment group was found.

### Planting ramets in the assay plots

Due to the disturbance that planting and watering a substantial number of ramets would have created in the on-going BioCON experimental plots, we used supplementary plots (assay plots) that were created in the BioCON rings in 1999 but that were not in use at the time of our study. These plots (1.5 m × 2 m, six per BioCON ring) were created within the FACE rings but on the edge of the main BioCON plots. Within each of the six BioCON rings, we chose three species-poor plots, consisting predominantly of *P. pratensis* (and from which we regularly weed out other species), and three species-rich plots, that best matched the original monoculture and 16-species plots respectively (Supplementary Fig. 4; Supplementary Methods 1). On 6–8 June 2012 ramets were planted into these 36 plots.

Ramets from all four CO_2_ × diversity source combinations were planted into each plot. As *P. pratensis* is rhizomatous, each plot was divided into quadrants (1 m × 0.75 m) by sinking sheet metal to a depth of 30 cm to prevent individuals from different selection environments interfering with one another. Within each quadrant, six to eight plants from the same selection environment, randomly drawn from among the mothers of the appropriate treatment type, were planted. Planted ramets were individually marked by loosely placing a piece of coloured wire around the base of each plant. To reduce transplant shock, the planted ramets were watered every day for the first two weeks, every second day for the third week, and every third day for the fourth week, after which watering was discontinued. Survival was high: 98.9% of individuals survived the first month. Ramets that died were not replaced.

### Measurement of plant performance

Transplanted ramets were grown in the field for two growing seasons. We assessed the survival of each ramet once per month between June and August in 2012 and 2013. In June 2013, we counted the number of inflorescences produced by each plant, and at the end of August 2013 all aboveground biomass was harvested, dried, and weighed. To estimate belowground biomass we measured root mass in a standardized volume of soil. Roots were cored by placing a 5 cm diameter PVC tube around the originally planted ramet and hammering it to a depth of 30 cm; all roots and soil contained in this core were extracted. This method was used instead of attempting to extract the entire root mass, due both to logistical constraints and because preliminary observations suggested little clonal expansion such that the majority of root growth would be captured within the cored sample. Consequently, we consider this to be a surrogate for root density (biomass per soil volume), rather than total root biomass, but for ease of reference we refer to this as ‘belowground biomass'. After the roots were extracted, they were washed free of soil and any roots obviously from a different species were removed. The roots were then dried and weighed.

Previous work has shown that total vegetative weight (stems, leaves and roots) and reproductive weight (fruits, surrounding glumes and rachis) are highly correlated in *P. pratensis*^45^. Similarly, amongst the plants that flowered, we find that the combined weight of fruits, glumes and rachis was highly correlated with both aboveground biomass (*P*<<<0.0001, adjusted *r*^2^=0.481) and total biomass (*P*<<<0.0001, adjusted *r*^2^=0.428). We thus consider biomass to be a rough proxy for fitness.

### Statistical analysis

*Analysis of biomass data*. We first tested for local adaptation to CO_2_ by holding the diversity environment constant; that is, by only including plants that were selected and assayed in the same diversity environment. Separate linear mixed effects models were performed on the logarithm (to meet assumptions of normality) of aboveground, belowground and total (above-+belowground) biomass. As fixed factors, we included all single and two-way interactions between the previous CO_2_ selection environment (CO_2_^sel^), the change in CO_2_ environment (ΔCO_2_) relative to the CO_2_ selection environment, and the general diversity environment (that is, either species-rich to species-rich or species-poor to species-poor). All models also included the following random effects: selection plot, family (mother's ID) nested within selection plot, assay ring and assay plot nested within assay ring. There was no statistical difference between the rings in which the plants were selected and much of this variance is likely absorbed by the term selection plot; thus we excluded selection ring from all models. We implemented all final models using the restricted maximum-likelihood method (REML) to estimate variance components^46^. The significance of all fixed effects was evaluated using type III estimable functions, and denominator degrees of freedom were determined using Satterthwaite's approximation^47^.

Testing scenario one: To determine whether local adaptation occurred regardless of diversity environment we performed separate linear mixed effects models on the logarithm of above-, belowground and total biomass while averaging over diversity environment. Analyses were performed as described above except that the performance of plants was averaged over the assay diversity environment (div^ass^), while selection diversity environment (div^sel^) was included in the model instead of the general diversity environment.

Testing scenarios two, three and four: We performed separate linear mixed effects models on the logarithm of aboveground, belowground and total biomass. As fixed factors, we included the previous CO_2_ selection environment (CO_2_^sel^) and diversity selection environment (div^sel^), which together define the BioCON plot from which each individual's mother was sampled. We also included fixed-factor terms indicating whether the plants experienced a change in CO_2_ (ΔCO_2_) or in diversity (Δdiv), relative to the plots of their mothers. As an alternative statistical approach, we also analysed the data by treating the selective and assay (current) environments (CO_2_^ass^ and div^ass^) (not the change in environments) as fixed factors. All models also included the following random effects: selection plot, family (mother's ID) nested within selection plot, assay ring and assay plot nested within assay ring into which ramets were transplanted. For the reasons outline above selection ring was excluded from all models.

To test for our hypothesized interactions we ran each full model and eliminated non-significant terms using likelihood ratio tests until the model contained the CO_2_^sel^ × ΔCO_2_ × div^sel^ (scenario two and three) and CO_2_^sel^ × ΔCO_2_ × Δdiv (scenario four) interactions and all lower terms. If a different three-way or the four-way interaction was significant then this was retained in the model and lower terms were not eliminated. The fit of each final model was assessed through visual inspection of the fitted and residual values, all models were found to meet assumptions of normality and homogeneity^46^. Data analysis was carried out in R version 3.0.2 (ref. 48) using the lme4^49^ and lmerTest^50^ packages. The analysis of biomass was carried out on all individuals that survived to the end of the experiment. However, data from 49 individuals that were growing in two plots that burned in a fire in May 2013 (three quarters of the individuals in one species-poor plot and all individuals in one species-rich plot in eCO_2_) and 34 plants that could not be relocated in spring 2013 were excluded. Thus in total, data for 765 individuals were included for the biomass analyses. In addition, 11 belowground biomass samples were excluded from the analysis as these cores were either hammered in at an angle that missed most of the root mass during the core extraction process or half the soil fell out of the core during extraction. We implemented all final models using the restricted maximum-likelihood method to estimate variance components^46^. The significance of all fixed effects was evaluated using type III estimable functions, and denominator degrees of freedom were determined using Satterthwaite's approximation^47^.

*Analysis of survival and inflorescence production*. To assess the importance of CO_2_^sel^, ΔCO_2_, div^sel^ and Δdiv on survival and inflorescence production we used aster models with random effects^51^ implemented in R^48^. Aster models facilitate the analysis of multiple life history stages as they can analyse survival and reproduction jointly. Furthermore, different life history traits can be modelled with different probability distributions and account for the fact that later components of fitness (for example, flowering) depend on earlier components of fitness (for example, survival)^24^. In our Aster model we included (1) survival until the time of inflorescence production in June 2013 (Bernoulli), (2) whether a surviving plant produced inflorescences (Bernoulli) and (3) the number of inflorescences produced (zero-truncated Poisson). The influence of each fixed factor was tested by comparing models with a given factor to a model containing only the random effects (null model) using log-likelihood ratio tests. Similarly the significance of each two-way, three-way and four-way interaction was tested individually against a reduced model that contained all lower terms. Selection plot, maternal family and assay plot were included as random effects. Analyses were carried out in the same order as described above for biomass: that is, we firstly tested for local adaptation holding diversity environment constant, then we tested for local adaptation averaging across the diversity assay environments, and lastly, we tested scenarios two, three and four. We also confirmed our results by re-running all models using CO_2_^cur^ and div^ass^ instead of ΔCO_2_ and Δdiv (Supplementary Note 1; Supplementary Table 6). From the Aster analysis, we excluded 17 plants that died within the first month after transplantation (June 2012) because death was more likely to be due to transplant shock than any other factor of interest, as well as the 8 individuals that could not be relocated in the spring of 2013 and the individuals that were in the two burned plots.

### Data availability

The data that support the findings of this study are available from the Dryad Digital Repository: DOI: doi:10.5061/dryad.s4bt7 (ref. 52).

## Additional information

**How to cite this article:** Kleynhans, E. J. *et al.* Adaptation to elevated CO_2_ in different biodiversity contexts. *Nat. Commun.* 7:12358 doi: 10.1038/ncomms12358 (2016).

## Supplementary Material

Supplementary Information Supplementary Figures 1-4, Supplementary Tables 1-9, Supplementary Notes 1-2, Supplementary Methods and Supplementary References
