# Supplementary material for: Adaptation to elevated CO2 in different biodiversity contexts
Source: Nat Commun. 2016 Aug 11;7:12358. doi: 10.1038/ncomms12358 (PMC4987528; doi:10.1038/ncomms12358)
Supplement: Supplementary Information — Supplementary Figures 1-4, Supplementary Tables 1-9, Supplementary Notes 1-2, Supplementary Methods and Supplementary References [file ncomms12358-s1.pdf]

Supplementary Information

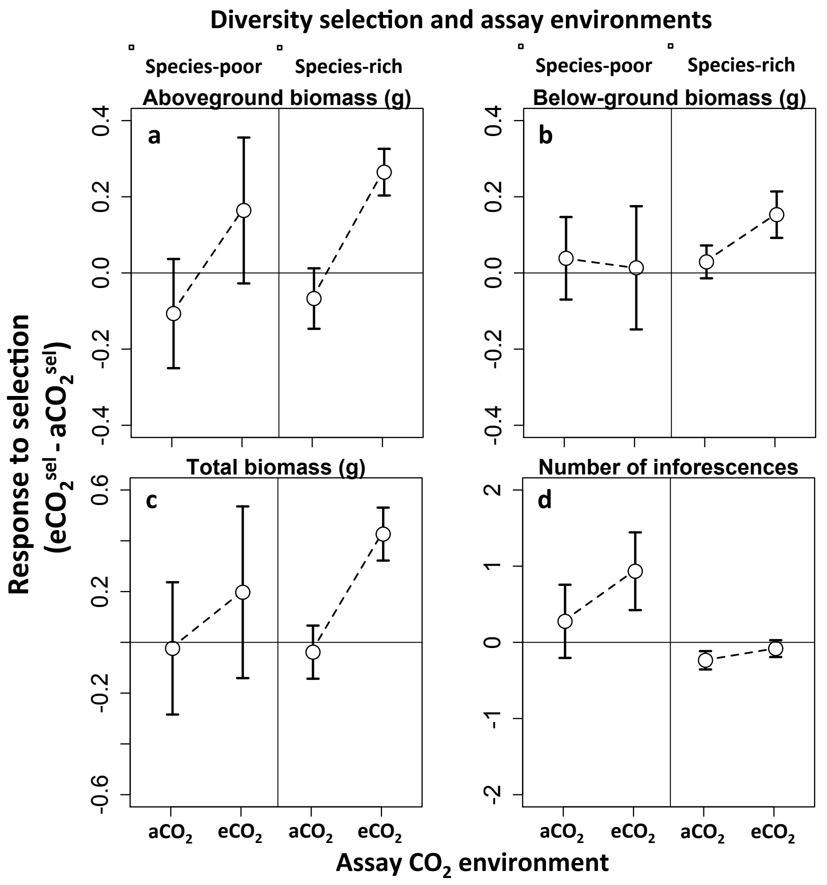

Supplementary Figure 1: Local adaptation of *P. pratensis* to elevated CO_2 when holding diversity environment constant for **a**, aboveground biomass, **b**, belowground biomass, **c**, total biomass and **d**, number of inflorescences. For each CO_2 assay environment we calculated the difference in biomass (g) or number of inflorescences (±1 standard error of the mean (s.e.m)) produced by plants that had previously experienced selection in eCO_2 and in aCO_2 from the raw data. Note the data points in this figure are the same as in figure 3 except that points for individuals that were selected and assayed in different diversity environments have been removed.

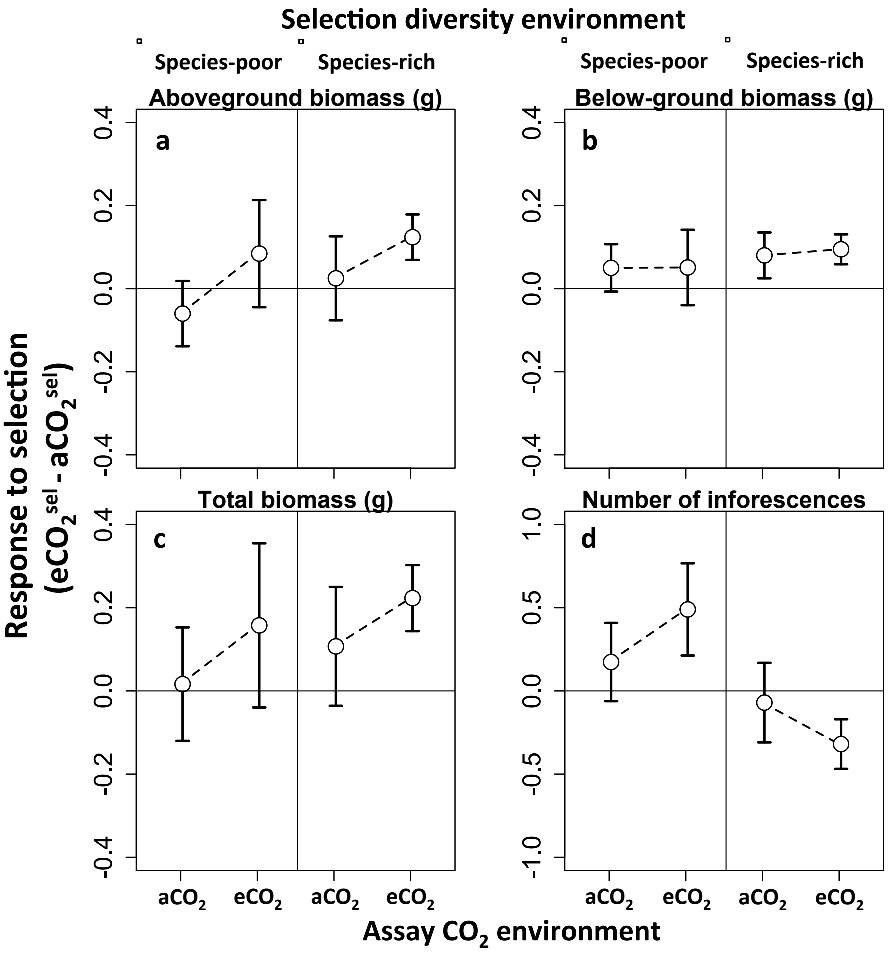

Supplementary Figure 2: Adaptation of *P. pratensis* to elevated CO_2 when selected in communities of low and high species richness averaged across diversity assay environments for **a**, aboveground biomass, **b**, belowground biomass, **c**, total biomass and **d**, number of inflorescences. For each CO_2 assay environment we calculated the difference in biomass (g) or number of inflorescences (±1 s.e.m) produced by plants that had previously experienced selection in eCO_2 and in aCO_2 from the raw data. Both aboveground and total biomass showed patterns consistent with adaptation to eCO_2, where there was greater biomass in the presence of eCO_2 for plants that had previously experience eCO_2 with the same holding for plants exposed to aCO_2 (positive dashed slopes). The trends were not significant, however, when ignoring the current community, as done here.

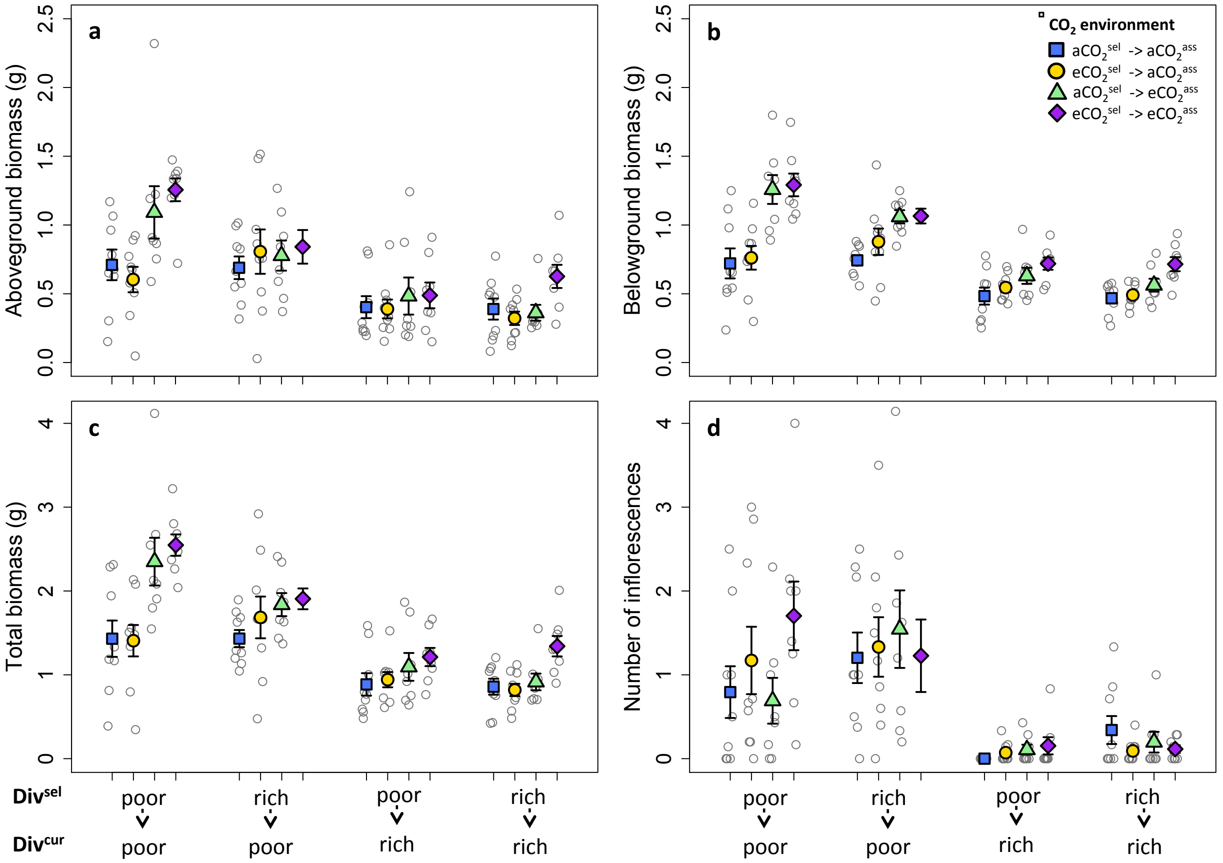

Supplementary Figure 3: Average **a**, aboveground biomass, **b**, belowground biomass, **c**, total biomass and **d,** number of inflorescences produced by plants originating from each of the selection environments (CO_2 and diversity) and grown in each of the assay environments (CO_2 and diversity). Open circles represent the mean of plants sampled from the same CO_2 and diversity environment and grown in the same assay plot. The black filled symbols are the mean (±1 s.e.m.) biomass and number of inflorescences averaged across all the plots in a treatment from the raw data

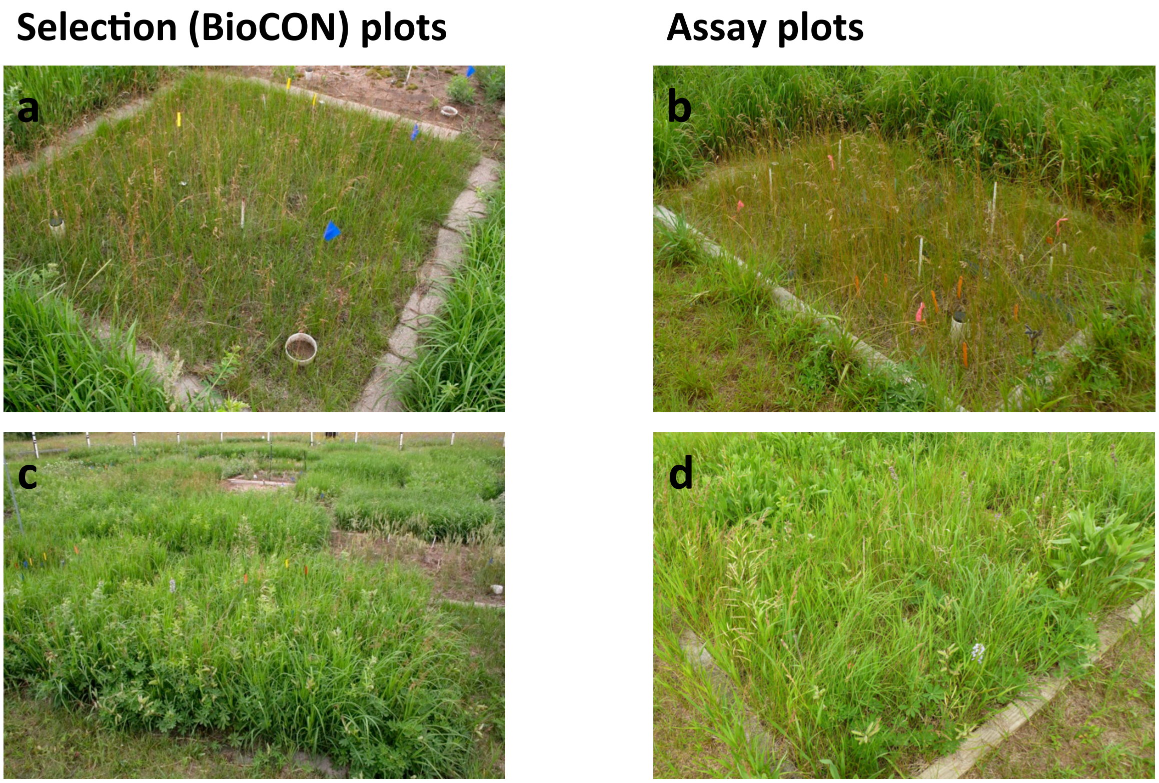

Supplementary Figure 4: Photographs of the original selection plots (BioCON plots) (left) and the assay plots (transplant plots) used in the experiment (right) with either *P. pratensis* dominated species-poor **a, b,** or species-rich plots **c, d** (photos taken by EJK).

Supplementary Table 1: Model output for analysis of aboveground, belowground, total biomass and survival and inflorescence data, when diversity environment is held constant i.e. plants selected in low diversity were assayed in low diversity. Biomass data was log transformed and analysed using linear mixed effects models, with F-tests for fixed effects constructed in R and denominator degrees of freedom (df_D) obtained from the Satterthwaite approximation. Significance of random effects was determined by likelihood ratio tests. Survival and number of inflorescences was determined using aster models to account for dependence among fitness estimates. The significance of each factor was evaluated using a likelihood ratio test. Note that the significant $CO_2^{sel} \times \Delta CO_2$ term is a consequence of significant CO_2^{ass} effects (see Supplementary Table 4) and are measures of a plastic response to the assay environment.

		**Aboveground biomass (g)**		**Belowground biomass (g)**		**Total biomass (g)**		**Survival & number of inflorescences**	
	df	df_D	F value	df_D	F value	df_D	F value	Test df	Test Deviance
CO_2^{sel}	1	11.1	0.1	6.0	1.6	55.7	1.0	1	0.1
ΔCO_2	1	306.6	7.8**	301.8	0.5	302.9	4.1*	1	0.4
div	1	12.2	23.9**	6.9	46.8***	42.0	46.0****	1	14.6***
$\Delta CO_2 \times$ div	1	306.4	0.1	301.6	2.0	302.7	1.1	1	0.9
$CO_2^{sel} \times \Delta CO_2$	1	3.7	4.7.	3.6	12.2*	3.7	7.6.	1	0.2
$CO_2^{sel} \times$ div	1	11.1	3.2	6.1	1.0	55.7	2.3	1	7.6**

**Random effects**	n	P	p	p	p
Selection plot	20	ns	ns	ns	0.02
Mother nested in selection plot	64	0.0006***	0.01*	0.0001***	0.005**
Assay ring	6	ns	ns	ns	-
Assay plot nested in ring	35	ns	ns	ns	0.0006***

ns P > 0.1; † P < 0.1; * P < 0.05, ** P < 0.01, *** P < 0.001, **** P < 0.000

Supplementary Table 2: Model output for analysis of aboveground, belowground, total biomass and survival and inflorescence data, when average over the diversity assay environment. Biomass data was log transformed and analysed using linear mixed effects models, with F-tests for fixed effects constructed in R and denominator degrees of freedom (df_D) obtained from the Satterthwaite approximation. Significance of random effects was determined by likelihood ratio tests. Survival and number of inflorescences was determined using aster models to account for dependence among fitness estimates and the significance of each factor was evaluated using a likelihood ratio test. Note that the significant $CO_2^{sel} \times \Delta CO_2$ term is a consequence of significant CO_2^{ass} effects (see Supplementary Table 4) and are measures of a plastic response to the assay environment.

		**Aboveground biomass (g)**		**Belowground biomass (g)**		**Total biomass (g)**		**Survival & number of inflorescences**	
	df	df_D	F value	df_D	F value	df_D	F value	Test df	Test Deviance
CO_2^{sel}	1	59.6	0.2	59.1	2.6	60.3	1.7	1	0.9
ΔCO_2	1	682.6	2.5	667.6	0.05	671.0	1.1	1	2.5
div^{sel}	1	59.9	0.9	59.5	0.7	60.7	0.7	1	2.2
$\Delta CO_2 \times div^{sel}$	1	681.8	0.003	666.1	0.3	670.3	0.2	1	0.9
$CO_2^{sel} \times \Delta CO_2$	1	3.5	3.6	32.7	10.7**	32.9	7.8**	1	0.7
$CO_2^{sel} \times div^{sel}$	1	59.6	3.0†	59.1	0.5	60.3	1.4	1	2.4
Random effects	n	p		p		p		p	
Selection plot	20	ns		ns		ns		ns	
Mother nested in selection plot	64	<0.0001****		<0.0001****		<0.0001****		<0.0001****	
Assay ring	6	ns		ns		ns		-	
Assay plot nested in ring	35	<0.0001****		<0.0001****		<0.0001****		<0.0001****	

ns P > 0.1; † P < 0.1; * P < 0.05, ** P < 0.01, *** P < 0.001, **** P < 0.000

Supplementary Table 3: Linear mixed effects model analysis of log-transformed aboveground, belowground and total biomass data. F-tests for fixed effects were constructed in R, with denominator degrees of freedom (df_D) obtained from the Satterthwaite approximation. Significance of random effects was determined by likelihood ratio tests. Note that the significant $div^{sel} \times \Delta div$ and $CO_2^{sel} \times \Delta CO_2$ terms are a consequence of significant div^{ass} and CO_2^{ass} effects (see Supplementary Table 4) and are measures of a plastic response to the assay environment.

		**Aboveground biomass (g)**		**Belowground biomass (g)**		**Total biomass (g)**	
	**df**	**df_D**	**F value**	**df_D**	**F value**	**df_D**	**F value**
CO_2^{sel}	1	59.8	0.2	59.4	2.3	60.4	1.6
div^{sel}	1	60.3	0.9	59.8	1.0	60.7	0.9
ΔCO_2	1	680.0	2.8	675.1	0.01	671.9	1.3
Δdiv	1	686.9	0.01	687.3	0.9	681.3	0.002
$CO_2^{sel} \times \Delta CO_2$	1	3.7	3.7	3.9	13.7*	3.8	9.4*
$div^{sel} \times \Delta div$	1	24.4	32.4****	20.3	99.5****	24.1	100.4****
$CO_2^{sel} \times div^{sel}$	1	59.9	3.0†	59.5	0.3	60.5	1.4
$\Delta CO_2 \times div^{sel}$	1	680.0	0.03	675.0	0.5	672.3	0.1
$CO_2^{sel} \times \Delta div$	1	683.0	0.01	679.7	0.1	675.1	0.01
$\Delta CO_2 \times \Delta div$	1	683.7	4.8*	681.0	0.5	676.6	3.3†
$CO_2^{sel} \times \Delta CO_2 \times \Delta div$	1	689.1	5.8*	690.3	4.2*	684.3	4.2*
$CO_2^{sel} \times \Delta CO_2 \times div^{sel}$	1	687.5	1.4	686.0	1.2	681.3	1.6
**Random effects**	n	P		P		P	
Selection plot	20	ns		ns		ns	
Mother nested in selection plot	64	P<0.0001****		P<0.0001****		P<0.0001****	
Assay ring	6	ns		ns		ns	
Assay plot nested in ring	35	0.0001***		ns		0.07†	

ns P > 0.1; † P < 0.1; * P < 0.05, ** P < 0.01, *** P < 0.001, **** P < 0.000

Supplementary Table 4: Linear mixed effects model analysis of log-transformed aboveground, belowground and total (aboveground + belowground) biomass data. F-tests for fixed effects were constructed in R, with denominator degrees of freedom (df_D) obtained from the Satterthwaite approximation. Significance of random effects was determined by likelihood ratio tests. Selection and assay environments are indicated as "sel" and "ass" respectively.

		**Aboveground biomass (g)**		**Belowground biomass (g)**		**Total biomass (g)**	
	**df**	**df_D**	**F value**	**df_D**	**F value**	**df_D**	**F value**
CO_2^{sel}	1	62.3	1.0	62.3	0.4	62.8	0.0
div^{sel}	1	60.2	0.9	59.8	1.0	60.7	0.8
CO_2^{ass}	1	5.7	5.0†	6.1	14.5**	6.0	10.8*
div^{ass}	1	51.7	20.2****	66.5	44.9****	73.5	51.4****
$CO_2^{sel} \times CO_2^{ass}$	1	676.6	1.4	674.1	0.4	670.3	0.2
$div^{sel} \times div^{ass}$	1	684.5	0.0	685.2	0.8	678.8	0.02
$CO_2^{sel} \times div^{sel}$	1	59.9	3.0†	59.6	0.3	60.5	1.4
$CO_2^{ass} \times div^{sel}$	1	684.4	1.4	683.6	1.2	678.4	1.6
$CO_2^{sel} \times div^{ass}$	1	679.1	0.5	678.2	0.4	672.1	0.4
$CO_2^{ass} \times div^{ass}$	1	52.1	2.8	67.1	3.2†	74.6	3.2†
$CO_2^{sel} \times CO_2^{ass} \times div^{ass}$	1	681.9	1.1	680.9	0.2	670.0	0.2
$CO_2^{sel} \times div^{sel} \times div^{ass}$	1	680.3	0.02	677.8	0.1	672.8	0.03
$CO_2^{sel} \times CO_2^{ass} \times div^{sel}$	1	677.5	0.03	673.1	0.5	676.1	0.2
$CO_2^{ass} \times div^{sel} \times div^{ass}$	1	685.4	6.0*	686.5	4.3*	680.4	4.4*
$CO_2^{sel} \times CO_2^{ass} \times div^{sel} \times div^{ass}$	1	681.1	4.5*	679.0	0.4	674.3	2.9†

**Random Effects**	n	P	P	P
Selection plot	20	ns	ns	ns
Mother nested in selection plot	64	P<0.0001****	P<0.0001****	P<0.0001****
Assay ring	6	ns	ns	ns
Assay plot nested in ring	35	0.0001***	ns	0.07†

ns P > 0.1; † P < 0.1; * P < 0.05, ** P < 0.01, *** P < 0.001, **** P < 0.0001

Supplementary Table 5: Summary of Aster model comparisons on *P. pratensis* survival and inflorescence production. Simpler models were tested against fuller models, with each term added independently (i.e., single terms were tested against the *Null* model, two-way interactions were tested against the Σ *Main effects* model etc.). Analysis of deviance (−2 log likelihood) and χ^2 test P-values are presented. Note that the significant $div^{sel} \times \Delta div$ term is a consequence of the significant div^{ass} effect (see Supplementary Table 6) and is a measures of a plastic response to the assay environment.

**Model Formula**	
Null:	Response = survival at preceding stage + No. inflorescences + random terms
Σ *Main effects*:	Response = survival at preceding stage + No. inflorescences × (CO_2^{sel} + ΔCO_2 + div^{sel} + Δdiv + random terms)
Σ *Two-way*:	Response = survival at preceding stage + No. inflorescences × (CO_2^{sel} + ΔCO_2 + div^{sel} + Δdiv + all two-way interactions + random terms)
Σ *Three-way*:	Response = survival at preceding stage + No. inflorescences × (CO_2^{sel} + ΔCO_2 + div^{sel} + Δdiv + all two-way interactions + all three-way interactions + random terms)

**Term**	**Residual d.f.**	**Test d.f.**	**Model deviance**	**Test deviance**	**P**
Null	5		-1935		
CO_2^{sel}	6	1	-1934	0.94	0.33
div^{sel}	6	1	-1932	2.21	0.14
ΔCO_2	6	1	-1931	3.14	0.08†
Δdiv	6	1	-1935	0.01	0.94
Σ *Main effects*	9		-1927		
$CO_2^{sel} \times div^{sel}$	10	1	-1925	2.18	0.14
$CO_2^{sel} \times \Delta CO_2$	10	1	-1927	0.60	0.44
$\Delta CO_2 \times div^{sel}$	10	1	-1926	0.69	0.41
$CO_2^{sel} \times \Delta div$	10	1	-1927	0.03	0.86
$div^{sel} \times \Delta div$	10	1	-1891	36.28	P<0.0001****
$\Delta CO_2 \times \Delta div$	10	1	-1924	2.95	0.09 †
Σ *Two-way*	15		-1880		
$CO_2^{sel} \times \Delta CO_2 \times div^{sel}$	16	1	-1880	0.50	0.48
$CO_2^{sel} \times div^{sel} \times \Delta div$	16	1	-1880	0.55	0.46
$CO_2^{sel} \times \Delta CO_2 \times \Delta div$	16	1	-1880	0.32	0.57
$\Delta CO_2 \times div^{sel} \times \Delta div$	16	1	-1880	0.14	0.71

Σ *Three-way*	19		-1876		
$CO_2^{sel} \times \Delta CO_2 \times div^{sel} \times \Delta div$	20	1	-1874	2.11	0.15

Random Terms					
Null	5		-1935		
Selection plot	5		-1935	0.04	0.42
Mother	5		-1959	24.20	P<0.0001****
Assay plot	5		-2075	140.15	P<0.0001****

ns P > 0.1†; P < 0.1; * P < 0.05, ** P < 0.01, *** P < 0.001, **** P < 0.0001

Supplementary Table 6: Summary of Aster model comparisons to test for differences in *P. pratensis* survival and inflorescence production as a result of the selection and assay CO_2 and diversity environments. Simpler models were tested against fuller models with each term added independently. Analysis of deviance (−2 log likelihood) and χ^2 test P-values are presented.

Model Formula	
Null:	Response = survival at preceding stage + No. inflorescences + random terms
Σ *Main effects*:	Response = survival at preceding stage + No. inflorescences × (CO_2^{sel} + CO_2^{ass} + div^{sel} + div^{ass} + random terms)
Σ *Two-way*:	Response = survival at preceding stage + No. inflorescences × (CO_2^{sel} + CO_2^{ass} + div^{sel} + div^{ass} + all two-way interactions + random terms)
Σ *Three-way*:	Response = survival at preceding stage + No. inflorescences × (CO_2^{sel} + CO_2^{ass} + div^{sel} + div^{ass} + all two-way interactions + all three-way interactions + random terms)

Term	Residual d.f.	Test d.f.	Model deviance	Test deviance	P
Null	5		-1935		
CO_2^{sel}	6	1	-1934	0.94	0.33
div^{sel}	6	1	-1932	2.21	0.14
CO_2^{ass}	6	1	-1934	0.58	0.45
div^{ass}	6	1	-1899	35.15	P<0.0001****
Σ *Main effects*	8		-1895		
$CO_2^{sel} \times div^{sel}$	9	1	-1893	2.25	0.13
$CO_2^{sel} \times CO_2^{ass}$	9	1	-1892	2.60	0.11
$CO_2^{ass} \times div^{sel}$	9	1	-1895	0.31	0.58
$CO_2^{sel} \times div^{ass}$	9	1	-1892	2.93	0.09†
$div^{sel} \times div^{ass}$	9	1	-1893	2.42	0.12
$CO_2^{ass} \times div^{ass}$	9	1	-1895	0.10	0.75
Σ *Two-way*	15		-1885		
$CO_2^{sel} \times CO_2^{ass} \times div^{sel}$	16	1	-1885	0.27	0.60
$CO_2^{sel} \times div^{sel} \times div^{ass}$	16	1	-1883	1.54	0.22
$CO_2^{sel} \times CO_2^{ass} \times div^{ass}$	16	1	-1884	1.27	0.26
$CO_2^{ass} \times div^{sel} \times div^{ass}$	16	1	-1881	3.66	0.06†
Σ *Three-way*	19		-1879		
$CO_2^{sel} \times CO_2^{ass} \times div^{sel} \times div^{ass}$	20	1	-1874	5.04	0.02*

<u>Random Terms</u>					
Null	5		-1935		
Selection plot	5		-1935	0.04	0.42
Mother	5		-1959	24.20	P<0.0001****

Assay plot	5	-2075	140.15	P<0.0001****

ns P > 0.1†; P < 0.1; * P < 0.05, ** P < 0.01, *** P < 0.001, **** P < 0.0001

Supplementary Table 7: GLM of aboveground biomass versus percent cover of species. Only species with an average percent cover greater than five percent in the assay plots were analyzed.

a) Selection in eCO_2 and species-rich and assayed in eCO_2 and species-rich plots

**Coefficients**	**Estimate**	**SE**	**F value**	**P**
intercept	-3.98	3.11		
Andropogon gerardii	-0.63	0.37	2.92	0.09
Bromis inermis	-0.00	0.79	0.00	0.99
Leptoloma cognatum	-0.71	0.49	2.11	0.15
Lupinus perennis	0.05	0.72	0.00	0.94
Panicum virgatum	-0.19	0.31	0.38	0.54
Poa pratensis	0.02	0.66	0.00	0.97

b) Selection in aCO_2 and species-rich and assayed in aCO_2 and species-rich plots

**Coefficients**	**Estimate**	**SE**	**F value**	**P**
intercept	-12.07	5.87		
Andropogon gerardii	-1.89	0.97	3.77	0.06
Bromis inermis	-0.74	0.59	1.58	0.22
Leptoloma cognatum	0.04	0.07	0.37	0.55
Lupinus perennis	-2.71	2.08	1.70	0.20
Panicum virgatum	-1.95	1.26	-2.40	0.13
Poa pratensis	1.99	1.84	1.16	0.29

Supplementary Table 8: GLM analysis of aboveground biomass and percent cover per functional group. All species were included in the analysis.

a) Selection in eCO_2 and species-rich and assayed in eCO_2 and species-rich plots

Coefficients	Estimate	SE	F value	P
intercept	0.08904	1.12206		
C4 grass	-0.41447	0.31578	1.72	0.2
C3 grass	0.39552	0.3958	1.0	0.32
Forb	0.15472	0.11377	1.85	0.18
Legume	0.24439	0.75023	0.11	0.74

b) Selection in aCO_2 and species-rich and assayed in aCO_2 and species-rich plots

Coefficients	Estimate	SE	F value	P
intercept	-2.56	0.51		
C4 grass	-0.77	0.40	3.76	0.06
C3 grass	-1.04	0.54	3.71	0.06
Forb	0.00	0.04	0.01	0.91
Legume	0.00	0.36	0.00	0.99

Supplementary Table 9: Percent cover of species grown in the assay and in the selection (BioCON) plots for both species-poor and species-rich communities.

	**Plot diversity**	**Assay plots**		**Selection plots (BioCON)**	
		**aCO_2**	**eCO_2**	**aCO_2**	**eCO_2**
Poa pratensis	Poor	46	49	43	42
Poa pratensis	Rich	18.9	24.8	9	10
Achillea millefolium	Rich	0.2	0.5	0	0.01
Agropyron repense	Rich	0.1	0.1	1	0.6
Amorpha canescens	Rich	0.1	0.4	6	4
Andropogon geradii	Rich	13.5	14.4	16	25
Anemone cylindrical	Rich	1.1	0.5	0	0.01
Asclepias tuberosa	Rich	0.4	0.3	0.2	0.2
Bouteloua gracilis	Rich	0	0	0.07	0.03
Bromis inermis	Rich	13.5	10.5	7	6
Koeleria cristata	Rich	0	0	0.08	0.1
Lespedeza capitata	Rich	2.9	4.4	15	8
Lupinus perennis	Rich	21.7	20.8	26	22
Petalostemum villosum	Rich	0	0	0.2	0
Schizachyrium scoparium	Rich	1.6	3.1	0.3	0.4
Solidago rigida	Rich	1.1	2.8	3	5
Sorghastrum nutans	Rich	0	0	0.07	0.001
*Leptoloma cognatum**	Rich	8.9	11.7	-	-
*Panicum vigatum**	Rich	6.9	7.9	-	-

* Indicates species not planted into the BioCON plots. In BioCON these species are actively weeded out of any plots in which they appear.

Supplementary Note 1: Analysis with assay environment instead of change in environment.

Here we present a more in depth analysis of our results and re-analyse our data using the assay environments (CO_2^{ass} and div^{ass}) (not the change in environments) as fixed factors

Single effects of diversity and CO_2 environments: P. pratensis plants were found to respond to the assay environment in which they were growing. Plants assayed in species-poor communities produced 91% more aboveground, 70% more belowground, and 79% more total biomass than plants assayed in species-rich plots (aboveground biomass: $F_{1, 51.7} = 20.2$, $P < 0.0001$; belowground biomass: $F_{1, 66.5} = 44.9$, $P<0.0001$; total biomass $F_{1, 73.5} = 51.4$, $P<0.0001$) (Supplementary Table 4). Assay plot richness also strongly influenced flowering: 41% of individuals planted into species-poor plots flowered while only 9% of individuals planted into species-rich plots flowered (Supplementary Fig. 3d and Supplementary Table 6, div^{cur} $P<0.0001$). These results likely reflect the increased intensity of competition in the multi-species plots (Supplementary Fig. 3). In our analysis with Δdiv this single effect manifested itself as a highly significant $div^{sel} \times \Delta div$ interaction (Supplementary Table 3, 5, $P<0.0001$).

P. pratensis also responded to the assay CO_2 environment. Plants assayed in eCO_2 produced 37% more belowground biomass ($F_{1, 6.1} = 14.5$, $P=0.009$) and 34% more total biomass ($F_{1, 6.0} = 10.8$, $P=0.02$) than plants assayed in aCO_2. However, this response was only marginally significant for aboveground biomass ($F_{1, 5.7} = 5.0$, $P=0.07$) (Supplementary Table 4) and not significant for flowering (Supplementary Table 6). Again in our analysis with ΔCO_2 this single effect manifested itself as a significant $CO_2^{sel} \times \Delta CO_2$ interaction (Supplementary Table 3).

In our analysis using selection and assay environments (instead of change in the environment), we also found a significant interaction between CO_2^{ass} × div^{sel} × div^{ass}, for aboveground biomass ($F_{1,685.4}$ = 6.0, P=0.015), belowground biomass($F_{1,686.5}$ = 4.3, P=0.038) and total biomass ($F_{1,680.4}$=4.43, P=0.036) (Supplementary Table 4) and a marginally significant interaction for inflorescence production (P=0.06; Supplementary Table 6). As illustrated in Supplementary Fig. 3a-c, plants that experienced selection in species-poor plots and were assayed in species-poor plots showed the greatest biomass, but this advantage was substantial only when also assayed in eCO_2. This suggests that eCO_2 serves to amplify or reveal adaptations to communities with low diversity.

Supplementary Note 2: Correlation between aboveground biomass and percent cover of species in the assay plot.

To assess whether the degree of adaptation observed in the high diversity plots was driven by diversity per se or by the abundance of a particular species, we tested, using a GLM, whether aboveground biomass production was correlated with the percent cover of any of the species in the assay plot when plants were selected and assayed in the same environment. We did not find that there was any one species that strongly predicted the evolutionary change in aboveground biomass in *P. pratensis*, although *Andropogon gerardii* (a dominant C_4 grass) had a marginally negative influence in both ambient and elevated CO_2 environments (Supplementary Table 7a and 7b). We performed the same test but using percent cover of functional groups, instead of species identity, and again found that no single functional group strongly predicted the response in aboveground biomass of *P. pratensis,* although C3 and C4 grasses had a marginally negative influence in aCO_2 (Supplementary Tables 8a and 8b).

Supplementary Methods: History and construction of experimental assay plots

Due to the disturbance that planting and watering a substantial number of ramets back into the original BioCON plots would have created, we used supplementary plots that were created in the BioCON rings in 1999 but that were not in use at the time of our study. These supplementary plots (1.5m × 2m, twelve per BioCON ring), were created within the FACE rings on the edge of the main BioCON plots. Of the supplementary plots, half had received additional nitrogen and were not used in the current study, leaving six plots in six rings (36 plots in total) (from now on referred to as assay plots). Briefly, the history of the assay plots is as follows: in 1999 eight pin oak (*Quercus ellipsoidalis*) acorns were planted per plot and any vegetation that managed to invade from the surrounding old field or from BioCON was allowed to establish[1]. In 2007 all 36 assay plots were tilled. The six assay plots on the northern side of each ring were then planted with 12 trees and kept free of any invading vegetation through regular weeding until 2011 when all trees were harvested and vegetation from the surroundings was allowed to re-establish. In contrast, the six assay plots on the southern side of each ring were seeded with 6 prairie species (*Andropogon gerardii*, *Bromis inermis, Lespedeza capitata, Lupinus perennis, Elymus canadensis* and *Panicum vigatum*), the first four of which are also present in the BioCON experiment. In 2008 the southern assay plots were weeded of all species not initially seeded, and in 2009 they were weeded of any flowering non-seeded species. After 2009 invading vegetation was allowed to establish.

As of 2012 (i.e., the initiation of our experiment), the dominant species of the previously treed assay plots was *P. pratensis,* such that these plots were very close ecological analogues of the *P. pratensis* monoculture plots in BioCON (e.g., they were comparable in abundance of *P.*

pratensis, Supplementary Table 9 and Supplementary Fig. 4a-b). We weeded out all other species and used these assay plots as the species-poor treatment. The three southern assay plots were comparable in species composition and abundance to the 16-species BioCON plots and were thus used as our species-rich treatment (Supplementary Table 9, Supplementary Fig. 4c-d). Although our assay plots were not the same as the plots in which selection took place, we still found a significant $CO_2^{sel} \times CO_2^{cur} \times div^{sel} \times div^{cur}$ interaction for aboveground and total biomass (Fig. 3a, c Supplementary Table 4) suggesting that plants grew best when planted back into the same environment they were selected in ('home' plot advantage). This supports our view that the assay plots were similar to their matched BioCON selection plots.

Supplementary References

1. Davis, M. A. *et al.* Elevated atmospheric CO2 : a nurse plant substitute for oak seedlings establishing in old fields. *Glob. Chang. Biol.* **13,** 2308–2316 (2007).
